# Low Power Wide Area Networks (LPWAN) at Sea: Performance Analysis of Offshore Data Transmission by Means of LoRaWAN Connectivity for Marine Monitoring Applications

**DOI:** 10.3390/s19143239

**Published:** 2019-07-23

**Authors:** Lorenzo Parri, Stefano Parrino, Giacomo Peruzzi, Alessandro Pozzebon

**Affiliations:** Department of Information Engineering and Mathematics, University of Siena, 53100 Siena, Italy

**Keywords:** LPWAN, LoRaWAN, IoT, marine monitoring, offshore data transmission

## Abstract

In this paper the authors discuss the realization of a Long Range Wide Area Network (LoRaWAN) network infrastructure to be employed for monitoring activities within the marine environment. In particular, transmission ranges as well as the assessment of parameters like Signal to Noise Ratio (SNR) and Received Signal Strength Indicator (RSSI) are analyzed in the specific context of an aquaculture industrial plant, setting up a transmission channel from an offshore monitoring structure provided with a LoRaWAN transmitter, to an ashore receiving device composed of two LoRaWAN Gateways. A theoretical analysis about the feasibility of the transmission is provided. The performances of the system are then measured with different network parameters (in particular the Spreading Factor—SF) as well as with two different heights for the transmitting antenna. Test results prove that efficient data transmission can be achieved at a distance of 8.33 km even using worst case network settings: this suggests the effectiveness of the system even in harsher environmental conditions, thus entailing a lower quality of the transmission channel, or for larger transmission ranges.

## 1. Introduction

Low Power Wide Area Networks (LPWANs) have been widely employed in the last years in the most disparate fields whenever a high coverage, low cost and low power pervasive monitoring infrastructure is required. Those characteristics are mandatory any time that such networks have to be deployed in harsh environments so as to limit maintenance interventions which may be too time consuming and additionally entailing high running costs. LPWANs have then proved to be crucial for the realization of Internet of Things (IoT) architectures, where a large quantity of devices are required to transmit and receive small amounts of data to Internet cloud infrastructures. LPWAN-based IoT architectures can be found in the most disparate application fields: while the Smart City scenario [1,2,3] is probably the most studied, several other LPWAN-based systems can be found in the literature. In particular, LPWAN networks have been used in healthcare scenarios for remote patient monitoring [4] as well as in smart energy management [5], they have been deployed on monumental historical buildings [6] and in industrial plants [7,8,9], analyzing their possible placement even in critical use-cases [10,11,12] or at remote sites [13,14,15].

The work standing behind this paper essentially falls within this last application scenario. Indeed, a tailor-made LPWAN based on the Long Range (LoRa) modulation implementing the LoRa Wide Area Network (LoRaWAN) protocol was set up in a marine environment in order to fulfill the objectives of a research and development project, which will be described later on (see Section 3), named *SeaFactory*. In particular, a sensor node was offshore installed testing two elevations (i.e., 3.5 m and 2.1 m above sea level) and transmitting data towards land where two LoRaWAN Gateways forwarded such information to a remote server via the Internet.

This paper is structured as follows: Section 2 exposes some related works on long distance LoRa links as well as on LoRa based networks deployed in harsh environments (e.g., water basins) while in Section 3 the proposed application scenario is described. Section 4 treats some theoretical concepts on wireless transmissions. Within Section 5 the validating field tests for the LoRaWAN network are presented and in Section 6 their results are highlighted and discussed. Finally, in Section 7 remarks and conclusions are brought up.

## 2. Related Works

LoRaWAN is a MAC layer communication protocol based on the LoRa technology. As its name hints, LoRa is a long distance modulation that is able to reach coverage ranges up to few kilometers in urban areas and up to tens in rural environments. Either the LoRa modulation and the LoRaWAN protocol were deeply dissected within a thorough survey [16] by firstly presenting both LoRa and LoRaWAN and then by comparing and reviewing a conspicuous amount of related references that were published within the last three years beforehand the publication of this survey. Finally, the study in Reference [16] also points out possible improvements and open challenges that could be faced by making use of these two technologies.

Several papers have already presented various solutions exploiting LoRa networks deployed in as much application scenarios testing sundry performances [17,18,19] (e.g., transmission distances, energy consumption, reliability, packets collision and so on). Some of these studies will be reported hereinafter focusing on the ones reporting long LoRa links for networks employed in severe contexts. However, LoRa transmission over seawater is a hot research topic at the moment hence the literature is still lacking.

The concept of harsh environment encloses plenty of case studies and industrial plants are one of these. Such a framework was treated in Reference [9] where a LoRa sensor node for fault detection of chemical emissions was implemented. For the sake of simplicity, sensor node wireless coverage was not actually tested within a plant. Hence, it was validated indoor within a structure that can be compared with an industrial plant due to its size and morphological characteristics. Indeed, it is a five floor building having a rectangular shape whose sides are 70 m and 100 m each enclosing a total surface of 7.000 m^2^ per floor. Moreover, each of them hosts many rooms separated by large concrete walls up to 1 m of width. Rooms are employed as scientific laboratories containing miscellaneous electromagnetic instrumentation. In addition, the entire building is fully covered with WiFi networks for Internet connection. Thus, this test-bed may be categorized as a harsh one due to both the elevated level of electromagnetic noise and the dense presence of obstacles. Performances, in particular data loss rates, were satisfactory even if a worst case scenario was set up since the receiver was placed in a corner of the third floor and the transmitter was tested at several spots, some of them were the opposite corners with respect to the receiver, across the five storeys. Therefore, the outcome of such a study suggested that comparable performances could have been reached in Line of Sight (LoS) conditions as this paper test scenario entails.

Some tests of LoRa links over urban and rural environments are reported within the conference paper [20] and the relative results, in terms of covered distance, are comparable with the ones presented in this paper. However, trial settings differ from those that will be listed in Section 5. The most important one is the application scenario: even though there are usually no large obstacles (e.g., buildings or mountains) offshore, transmissions over the sea are especially a tough challenge since seawater behaves as a huge ground plane thus causing odd reflection phenomena. Another dissimilarity resides in antennas installation, since those used in this work were positioned lower than that of Reference [20], and a further one is represented by different employed transmission parameters. As it will be reported later on (see Section 5), in this paper six Spreading Factors (SFs) were tested rather than only one as the authors of Reference [20] did, even if with a lower number of broadcast packets.

Continuing with dense urban contexts, in a previous work [3] the authors of this paper developed a LoRa sensor node prototype for the remote sensing of the filling level of trash bins. During the validation tests only 1.1 km transmission range was achieved within a city centre and no more than 2.7 km exploiting LoS conditions. Similar outcomes (i.e., 2.2 km and 2.5 km) were respectively achieved by the authors of Reference [21] and of Reference [22]. Each of the two groups of researchers set up tests on LoRa transmissions exploiting a comparable scenario with the one of article [3]. These results are pretty scant though if compared with the related ones of this paper where, to improve the performances of the communication channel, two diversity schemes (i.e., space and frequency) were simultaneously established. The former was achieved by setting up two Gateways whose antennas occupied different spatial locations; the latter was reached due to the fact that, conversely from the prototype presented in the former work [3], the firmware running on this sensor node implemented the LoRaWAN protocol which provides for frequency hopping amid different channels.

The authors of Reference [23] also tested performances of LoRa links in an extremely high density urban area and the authors focused on assessing complete loss free communication. They installed a multi-channel LoRaWAN Gateway at a pretty high elevation (i.e., 40 m above the ground) by exploiting an existing building and then they tested a sensor node at the ground level periodically transmitting while it was carried around by a walking person. The results were in contrast with what several technical reports aver: in such a test-bed, LoRaWAN proved to be capable of tracking a person walking at street level without packet losses only within a 200 m range from the Gateway and a total loss of transmission was experienced at 600 m.

In Reference [1] the Italian city of Padua, which is a less dense urban environment in comparison with the one of Reference [23], was exploited as application scenario. Understanding how many Gateways should be deployed throughout the city in order to ensure a full LoRa citywide coverage, with the target of realising an infrastructure for smart city contexts, was one of such paper aims. The first step was to evaluate the coverage capabilities of a single Gateway and, according to the desired specifications of the paper, it was found out that a single Gateway covered a zone having a radius of 1.2 km. Since Padua extends over an area of around 100 km^2^, then an amount of 30 Gateways should be disposed. Similar results in terms of coverage assured by a single Gateway were also achieved within the article [24] where a slight different application scenario was proposed. However, Reference [24] shares testing methodologies and procedures with Reference [1].

With regards to long LoRa links, References [25,26] put forward interesting clues validated by a common measurements setup exploiting an overhead location (i.e., a mountain) to install one of the two end links. While the deployment scenario described in this paper does not present such orographic properties, comparable results were achieved, in terms of correctly received packets, with the ones obtained by the authors of Reference [25]. For what concerns the covered distance, they achieved a longer link with respect to the one presented in this paper. Nevertheless, we experienced enough link margin during the tests suggesting that more extended distances might have been covered. Reference [26] deserves particular attention. Indeed, apart from exploiting LoS conditions and 60% clearance of the first Fresnel zone due to the possibility of making use of a mountain, an especially designed LoRa transceiver having enhanced characteristics, thus increased performances, was realized and employed for the tests. In so doing, the impressive covered distance of 112.5 km was obtained. On the other hand, the sensor node used in this paper to perform the validating measurements campaign is based on general purpose off-the-shelf components, and is then not optimized as that exploited within Reference [26].

### LoRa Transmission over Water Basins

This Subsection exposes related works on LoRa links in marine and lacustrine environments that a priori confirmed the feasibility of such broadcasting. Indeed, as it was said earlier on, telecommunications over water, especially over the sea, are a tough problem since water basins act as huge ground planes entailing odd reflection occurrences. Thus they can be labelled to all intents and purposes as harsh environments.

The conference article in Reference [27] shows a LoRa network to implement a sailing monitoring system. The system was tested on a lake by installing sensor nodes on board of sailboats whose antennas were placed at 1.5 m above the water level while the one of the Gateway was installed at 4 m above the water level. Such elevations are comparable with the ones tested in this paper. The big differences between our tests and the ones of the aforesaid paper are the exploited Industrial, Scientific and Medical (SM) band (868 MHz and 433 MHz respectively), the transmitter power output (in turn, 25 mW and 100 mW), the tested SFs (all the SFs and only three SFs severally) and the tested Bandwidths (BWs) (125 kHz for our tests while both 250 kHz and 500 kHz where selected by the authors of Reference [27]). Notwithstanding these dissimilarities, similar outcomes may be observed: we were able to cover longer distances though ensuring lower rates of received packets.

Saline lakes are a hybrid scenario between a freshwater lake and the sea. They are massively exploited to mine salt by means of brine pumps. Hence, a remote real-time monitoring for such machineries is required in order to prevent them from failures. What has just been described is exposed in Reference [28] where a group of researchers set up a LoRa based LPWAN to monitor brine pumps installed in brine wells. The entire system was tested on the field for a timespan of a month showing an effective and dynamic monitoring of those machineries. However, no more than 700 m were covered since longer distances were not requested for this application scenario.

LoRaWAN technology has been also under investigation to be employed for emergency conditions monitoring for ships [29]. Sensor nodes were placed on board of boats sending emergency notifications via LoRa to Gateways, which are installed ashore, by covering a 5.11 km distance. Then, the latter ones forward these information to a cloud service that enables the harbor police to be aware if an emergency event occurred offshore by consulting an Android mobile application. This scenario resembles the one proposed in this paper since sensor nodes were installed offshore, sending packets ashore to Gateways.

Another work on real-time tracking and monitoring of boats, in particular of the lightweight ones, is exposed in Reference [30]. The authors implemented a LoRa LPWAN architecture to retrieve data from ships and make them accessible to users by means of a data visualization platform. Once again, sensor nodes were installed offshore on board of boats whilst a Gateway was situated ashore on the roof of a building. System performances were evaluated during some training sessions for sailboats races within a port area which entailed various spots of non-LoS conditions. Indeed, the entire infrastructure was supposed to be exploited for the remote and real-time monitoring of sailboats races. Several transmission parameters were analyzed throughout different sets of trials and the maximum coverage range experienced within such a scenario was 4 km.

LoRaWAN networks may be exploited as well for the assessment of potential tidal energy in sites at risk like the Philippines [31]. By means of specially designed buoyant sensor nodes such energy was sensed and wirelessly sent towards land, covering the maximum distance of 14.6 km. While this transmission distance is larger than the one achieved in this paper, the residual link margin measured during our tests suggests similar results. The same conclusion can be drawn by considering Reference [17]. Indeed, LoRaWAN coverage experiments were performed both on land and sea: in the latter case, a range that was slightly less than 30 km was observed. However, pretty high packet loss ratios were recorded.

Finally, the authors of Reference [32] showed two sets of measurements testing LoS and non-LoS conditions and exploiting either the 434 MHz and the 868 MHz ISM bands. In LoS setup, both the bands were evaluated and 22 km range was assured with off-the-shelf components. On the other hand, during non-LoS setup, only the lower band was adopted and, by taking advantage of expensive high gain antennas, 28 km were covered. However, both the end points of the LoRa links were placed some tens of meters above the sea level thus allowing to exploit, in the LoS condition, the 60% clearance of the first Fresnel zone.

## 3. Application Scenario

A complete LoRaWAN network infrastructure for the remote monitoring of overboard sea farms was set up, in order to fulfill the objectives of the *SeaFactory* project, a strategic research and development project, funded by the Tuscany Region, Italy, whose aim is to realize, for the benefits of companies dealing with fish farming, a prototype of an operational control centre for the management and for the monitoring of several assets. These include infrastructures, transformation plants and miscellaneous instruments which are situated either on land and sea. Moreover, *SeaFactory* also aims to provide services, to manage and diffuse information to companies that are part of the chain of processing and distribution of fish, as well as towards consumers, having the purpose of allowing the traceability of the product and making it easily accessible by the establishment of the Fishing Passport (i.e., a sort of “*Identity Card*”) of the bred fish.

For this purpose, a sensor node sampling several environmental parameters (i.e., water temperature and salinity, turbidity, pH, chlorophyll, wave intensity and current speed, redox potential and nitrate concentration) is expected to be assembled on board of a seamark buoy floating in the proximity of the breeding cages located offshore Piombino, Italy, belonging to Agroittica Toscana SRL, an Italian aquaculture company. For the time being, only a temporary installation has been accomplished, for the testing of the infrastructure and for the assessing of its feasibility as well. Hence, for the mere scope of validating the link, a fixed length payload (i.e., 51 B) containing a test string was ashore transmitted on a periodic basis, by making use of the LoRaWAN protocol, while two Gateways were placed within the ashore plants of the aquaculture company plant in Piombino, Italy. They were in charge of either demodulating the signals and measuring both the Received Signal Strength Indicator (RSSI) and the Signal-to-Noise Ratio (SNR) and finally of forwarding these data and metadata to a remote server exploiting the Message Queuing Telemetry Transport (MQTT) protocol.

Summarizing, we will show the usability of a LoRaWAN network within the marine environment despite of the intrinsic harshness of the deployment scenario. At the same time, the feasibility of a long LoRa link will be proven without exploiting overhead spots for the installation sites of the antennas together with the employment of off-the-shelf products only. Moreover, the regional regulations the LoRaWAN protocol is subject to [33] will be fully abided.

## 4. Transmission Channel Analysis

This Section is devoted to provide a brief review of basic and fundamental concepts related to wireless links. Such notions will be applied in Section 5 for the application scenario and taking into account the hardware components that were adopted for the measurement campaign.

The outcome of wireless transmissions is mainly conditioned by two key factors: LoS between the transmitter and the receiver and enough link margin at the receiver side. The former ensures the best propagation thus enlarging the probability of a successful communication. Such chances are furthermore enhanced if the prolate ellipsoidal volume wrapping the direct LoS path between the transmitter and the receiver (i.e., the first Fresnel zone) is free from obstacles. On the other hand, enough link margin at the receiver side is fundamental for the feasibility of the transmission. In other words, it is mandatory in order to achieve the broadcast that the RSSI of the received signal is higher than the receiver sensitivity. This requirement is assessed by evaluating the link budget of the communication.

### 4.1. Link Budget

The link budget equation accounts for all of the losses and gains from the transmitter to the receiver along the communication channel. Hence, the simplest link budget equation, logarithmically expressed, is a superposition of terms as

(1) $$P_{RX}=P_{TX}+G_{A}-L$$

where $P_{RX}$ and $P_{TX}$ are in turn the received power and the transmitter power output both expressed in dBm, $G_{A}$ represents the antennas gains on either the sides of the communication channel expressed in dBi and *L* stands for all the losses the channel is affected expressed in dB. Therefore, the aforesaid link margin $L_{m}$ is promptly calculated as

(2) $$L_{m}=P_{RX}-S_{RX}$$

where $S_{RX}$ is the receiver sensitivity expressed in dBm.

For the sake of completeness, it has to be also underlined that there exists another crucial requirement that has to be usually guaranteed in order to fulfill a satisfactory transmission: the SNR limit. In general, apart from achieving enough link margin, a signal whose power is bigger than the one of the noise that was added on it during its transmission has to be received. Such a condition entails a positive SNR provided that it is logarithmically expressed or, alternatively, greater than 1 if the SNR is linearly expressed. However, LoRa modulation enables receivers to demodulate signals below the noise floor power level due to its robustness.

**Figure 1:**
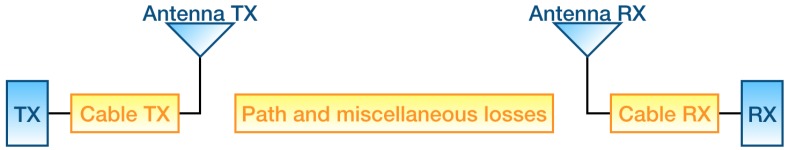
Gains, in blue, and losses, in yellow, of a communication channel to be accounted within link budget equation.

Coming back to link budget, a more detailed equation, though still simplified, can be derived by making reference to Figure 1 which displays a block diagram of an example of a communication channel:

(3) $$P_{RX}=P_{TX}+G_{TX}-L_{TX}-L_{FS}-L_{M}+G_{RX}-L_{RX}.$$

In Equation (3), $P_{TX}$ and $P_{RX}$ are the same of Equation (1), $G_{TX}$ and $G_{RX}$ respectively are the gains of the transmitting and receiving antennas expressed in dBi, while $L_{TX}$ and $L_{RX}$ are the losses due to cables and connectors severally at transmitter and receiver sides. $L_{FS}$ is the path loss and it is normally named as free space loss. Finally, $L_{M}$ represents miscellaneous losses due to manifold potential causes (e.g., antennas polarization mismatch, obstructions within the first Fresnel zone and so forth). Both the latter two losses are expressed in dB.

$L_{FS}$ in LoS propagation is the attenuation between the two antennas (i.e., the transmitting and the receiving ones) due to the distance between the extrema of the transmission link, considering that such a gap, better if the entire first Fresnel zone taken into account is free from obstacles. Such a loss can be expressed via different equations in function of the units of measure in which its composing terms are specified. A commonly adopted version is

(4) $$L_{FS}=32.45+20{log}_{10}\left(D\right)+20{log}_{10}\left(f\right)$$

where *D* is the distance between the transmitter and the receiver expressed in km and *f* is the transmission carrier frequency expressed in MHz.

### 4.2. Fresnel Zone

The Fresnel zone is an infinite series of confocal prolate ellipsoidal three-dimensional regions enveloping the direct LoS path between the transmitting and the receiving antennas. It is employed in propagation theory to evaluate losses due to reflection and diffraction between the endpoints of the wireless link. Such zones are usually called $F_{i}$ with $i\in N^{+}$ where $F_{1}$ is the smallest one. Regularly, a slight abuse of notation is committed since also the maximum radius relative to the ith zone is marked as $F_{i}$.

In order to grasp the practical meaning of the Fresnel zone, it has to be recalled that radio frequency waves propagate not only directly along the LoS path, but also in an off-axis fashion. This phenomenon entails reflections which in turn implies a phase variation on the reflected signal. Unfortunately though, if such unwanted and often unpredictable changes of phase amount to (2k+1)π with k∈N, then the direct and reflected signals cancel out at the receiver side since they have opposite phases. On the other hand, if the change of phase is equal to 2kπ with k∈N then the two signals will enhance each other at the receiver side due to the fact that they are perfectly in phase. However, reflections should be avoided since phase changing ordinarily leads to destructive interference and signal weakening.

A more rigorous definition is that the nth Fresnel zone is the locus of points belonging to the three-dimensional space such that a two-segment transmitted reflected signal, that deflects off a point on such a surface, will have a phase change equal to (i+1)π with respect to the signal propagated along the direct LoS path. Those zones are ellipsoids whose foci are the transmitter and the receiver antennas. In other words, even numbered Fresnel zones cause a phase change of π, thus damaging wave propagation, whilst odd numbered ones produce a phase change of 2π, wherefore intensifying the broadcast. Hence, despite an intuitive belief, LoS between transmitter and receiver is not theoretically sufficient to assure adequate wireless transmissions. Indeed, due to the complex nature of radio propagation, obstructions within the first Fresnel zone should be shunned since they can cause significant signal weakening consequently vanishing the enhancing effect of such a zone. Sadly though, it is almost unfeasible to obtain a completely empty $F_{1}$ so at least 60% clearance should be achieved [34].

**Figure 2:**
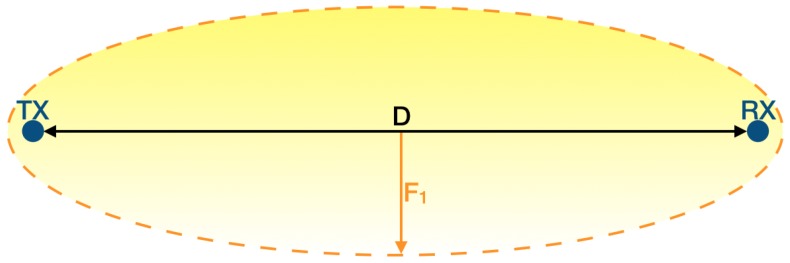
First Fresnel zone example.

Figure 2 reports an example of the first Fresnel zone: *D* is the distance between the transmitter and the receiver (i.e., the direct LoS path) while $F_{1}$ is the maximum radius of the zone which occurs at $\frac{D}{2}$. There exist formulas that evaluate $F_{1}$ depending on the units of measure in which its terms are expressed. If *D* is specified in km and the transmission frequency *f* is defined in GHz, then $F_{1}$ is calculated in m as

(5) $$F_{1}=8.656\sqrt{\frac{D}{f}}.$$

So far it was pointed out that achieving the direct LoS from the transmitter and the receiver along with a significant clearance of the first Fresnel zone is considerably beneficial to increase the chances of adequate wireless transmissions. For what matters the direct LoS, it is obtained by checking whether or not the receiving antenna is beyond the horizon with respect to the transmitting one. Fresnel zone clearance has to be assured not only by avoiding obstacles like buildings or mountains, but also by accounting for the Earth curvature bulge especially for long transmission distances. Both of these issues may be faced by overhead installing the antennas.

**Figure 3:**
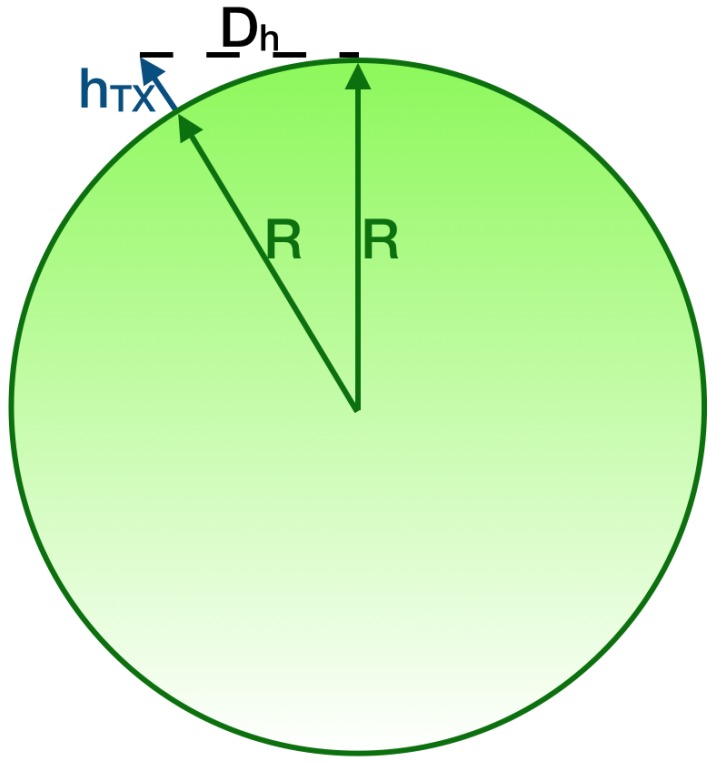
Evaluation of the distance from the horizon.

The direct LoS assessment considering the distance of the horizon from the transmitting antenna may be simply performed by making use of the Pythagorean theorem. Let approximate the Earth as a perfect sphere, having radius R=6371 km, with no terrain irregularities: such hypothesis is a suitable approximation when links take place over the sea. Moreover, suppose that the transmitting antenna is mounted at an altitude $h_{TX}$ above sea level (see Figure 3 bearing in mind that it is not in scale since its purpose is merely qualitative). Then, the distance from the horizon $D_{h}$ expressed in *m*, provided that either *R* and $h_{TX}$ are so, is

(6) $$\begin{array}{c} D_{h}=\sqrt{{\left(R+h_{TX}\right)}^{2}-R^{2}}=\sqrt{h_{TX}^{2}+2Rh_{TX}}. \end{array}$$

**Figure 4:**
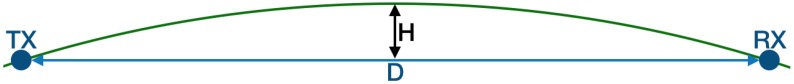
Example of the height of the Earth bulge in transmission links.

Earth curvature should be taken into consideration whenever long distance links (i.e., more than few kilometers) are designed. In particular, the bulge amid the two endpoints could obstruct the first Fresnel zone. The maximum Earth bulge height *H* expressed in m, which is experienced in the midpoint of the link (see Figure 4 bearing in mind that it is not in scale since its purpose is merely qualitative), is computed as

(7) $$H=\frac{1000D^{2}}{8kR}=\frac{125D^{2}}{\frac{4}{3}R}=\frac{375D^{2}}{4R}$$

where *D* is the distance between the endpoints expressed in km, *R* is the Earth radius and *k* is a factor which accounts for the bending effect on radio waves caused by the declining of atmospheric pressure. Practically speaking, this phenomenon implies an enlargement of the Earth radius by factor *k*. Actually, *k* also depends on weather conditions, but it is averagely equal to 4/3.

## 5. Data Transmission Tests

The performances of the LoRaWAN network infrastructure for the *SeaFactory* project were validated by sorting out a measurements campaign hosted by the Agroittica Toscana SRL aquaculture firm. In particular, the Gateways were installed in the ashore plant owned by that company while the end node was placed on board of a boat with which the closeness of the offshore cages was reached. There, several packets were transmitted, by employing six different SFs (i.e., 7,8,⋯,12), and their data and metadata were sampled. Moreover, the Coding Rate (CR) was set to 4/5 and a bandwidth of 125 kHz was exploited.

### 5.1. Test String Selection

As it was claimed earlier on (see Section 3), for the sake of simplicity, a test string was contained within the packet payload transmitted by the end node rather than the actual measurements furnished by sensors although, in a successive phase of the project, it is expected to include such data encoded into a string.

According to the LoRaWAN regional regulations [33], the packet payload has a variable maximum length which depends on the exploited SF to broadcast it. In order to limit the number of involved variables within the experiment, the test string was set to have the fixed length of 51 B since it is the minimum amid the aforesaid maximum sizes. Moreover, the aforementioned laws dictate rules on the temporal occupation of the band. Indeed, the adopted ISM band upon which the sensor node transmits signals cannot be utilised more than 1% of the time in order to allow as many devices as possible to make use of it.

### 5.2. End Node Installation

**Figure 5:**
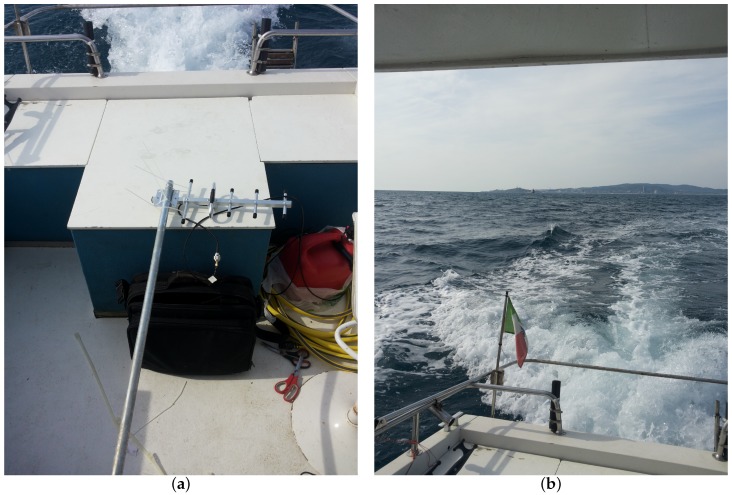
Offshore setup for the measurements campaign: (**a**) offshore end node antenna and its pole; (**b**) view approaching the tests spot.

The end node was set up bearing in mind that in future stages of the project it is expected to be mounted on the cages anchoring buoys (i.e., seamark buoys). A buoy like these is constructed as a beam, that has a truncated cone situated at one fourth from its lower extremity that allows for the buoyancy property, whose upper end is at 3.5 m above sea level. Therefore, the transmitting antenna will be placed at such height by directly installing it on the beam. However, for these measurements campaign it will be temporarily mounted on a pole feigning the aforesaid altitude above sea level. Actually, as it will be seen later on, two different heights for the transmitting antenna were tested in order to check the feasibility of the link simulating a worst case scenario. Hence, the transmitter was firstly at an altitude $h_{TX_{1}}=3.5$ m and then at an altitude $h_{TX_{2}}=2.1$ m above sea level. Moreover, the end node with the pole supporting the antenna was set up on board of a boat. Figure 5 displays the antenna installed on the pole lying on the floor of the boat and the view reaching the measurements spot.

So as to enhance the strength of the wireless link, a directional Yagi-Uda antenna having a gain $G_{TX}=9$ dBi was selected. Another interesting characteristic is that it is pretty lightweight (i.e., 400 g) thus well suiting to its future employment on top of a seamark buoy. Indeed, its limited weight could prevent the buoy capsizing. The antenna was connected to a B-L072Z-LRWAN1 discovery kit board produced by STMicroelectronics via a 4 m long coaxial cable whose overall loss, $L_{TX}=2.10$ dB, was measured by means of a vector network analyzer.

Eight different channels belonging to the 868 MHz ISM band were exploited to conduct the transmission tests so as to establish a frequency diversity scheme. Therefore, the B-L072Z-LRWAN1 discovery kit board performs a frequency hopping in a pseudo-random fashion for each transmission by switching amid the channels having the following carrier frequencies: 867.1 MHz, 867.3 MHz, 867.5 MHz, 867.7 MHz, 867.9 MHz, 868.1 MHz, 868.3 MHz and 868.5 MHz.

Finally, so as to speed up the tests, 6 discovery kit boards were sorted out. They transmitted the string that was introduced in Section 5.1 respectively employing a different SF. All those boards were supplied by the boat cigarette lighter.

### 5.3. Gateways Installation

Earlier on, in Section 3, it was claimed that two Gateways would be employed. Indeed, such a choice was made so as to improve the reliability of the link by establishing a space diversity scheme at the receiver side. In addition to it, LoRaWAN protocol itself provides for a time diversity scheme, that is ensured by the laws on temporal occupancy of the transmission band [33], and for a frequency diversity scheme, that is implemented by switching the carrier frequency in a pseudo-random fashion for each transmission. In so doing, a more robust broadcast is achieved.

The Gateways along with their antennas were ashore installed on top of a knoll, whose altitude is 6 m above sea level, which belongs to the proprietary terrain of the aquaculture partner company. With a view of better exploiting the first Fresnel zone, the receiver antennas were placed on a 7.2 m pole raised on top of the knoll. In particular, they were installed on the pole upper extreme by mounting them on a 2 m bar fastened to the pole forming a cross. Hence, by summing up the altitude of the knoll and the length of the mounting pole, the receiver antennas were located at a height of $h_{RX}=13.2$ m above sea level. As it will be seen later on, such a set up was far from being sufficient to allow for the 60% clearance of the first Fresnel zone, but, in order to keep the installation procedures as simple as possible, the antennas could not be placed any higher since only a temporary installation was planned for these early days of the validating experimentation phases of the project.

**Figure 6:**
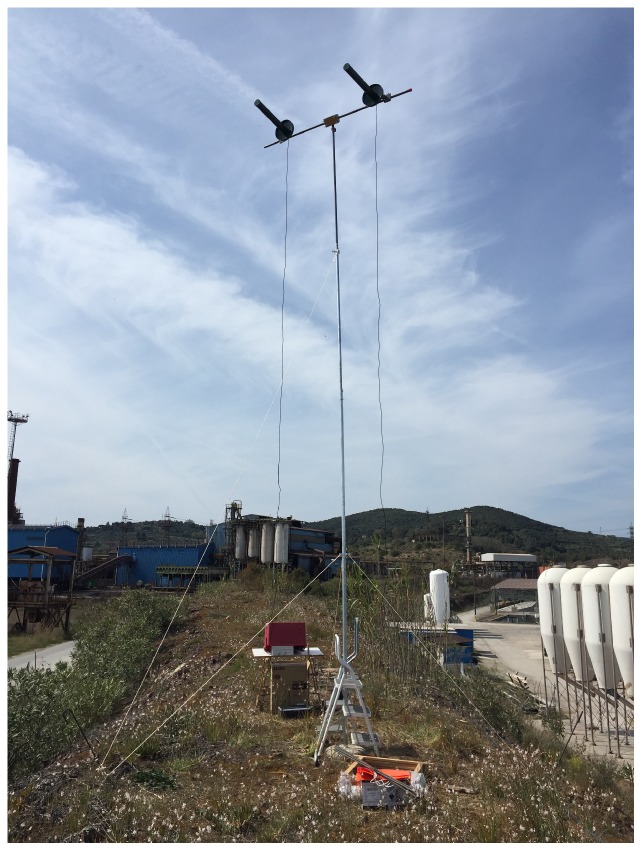
Ashore setup of the Gateways.

The Gateways were implemented by means of a RAK831 produced by the RAKWireless, which is a multi-channel LoRaWAN concentrator, driven by a Raspberry Pi 3 model B. Figure 6 shows the Gateways setup on top of the knoll.

Two directional helical antennas having a gain $G_{RX}=14$ dBi were selected for the Gateways. They were in turn connected to the Gateways via two 15 m long coaxial cables having a total loss of $L_{RX}=4.06$ dB each, that was measured by exploiting a vector network analyzer. Finally, Internet connectivity was guaranteed by a 4G LTE router since there were no other opportunities in loco. This slightly slowed down the communication between the Gateways and the network server introducing a minimal amount of latency: nevertheless it was not an issue at all.

### 5.4. Measurements

**Figure 7:**
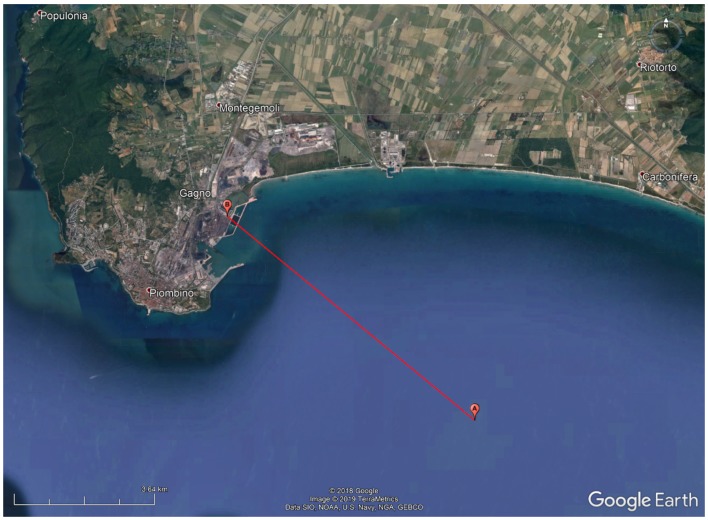
Map showing the positions of the end node, point A, and of the Gateways, point B along with the covered distance, red line.

The tests were performed from offshore, by reaching the closeness of the breeding cages, towards land covering the distance D=8.33 km. Figure 7 reports the position of the end node (i.e., point A), the position of the Gateways (i.e., point B) and the length of the link (i.e., the red line), whilst Table 1 lists the coordinates of points A and B.

The tests were accomplished with smooth sea conditions having a maximum wave height of 50 cm. It was a sunny day with a mean temperature of 18 °C and a gentle breeze whose average speed was of 4 m/s. Testing the system with more severe marine and weather conditions would have been extremely interesting. Unfortunately, though, the boat we had available for the fullfilment of the tests could not cast off in such cases. However, as it will be explained later on (see Section 6.2), sub-GHz wireless links (e.g., the ones exploiting LoRa modulation) are robust to these sorts of effects. Moreover, 600 packets were transmitted by respecting the laws on temporal occupation of the transmitting band [33] in an overall amount of time of approximately 14 h.

The measurements campaign was sorted out in 2 groups, namely #1 and #2, by covering the same link from point A to point B (see Figure 7) and by testing all the SFs by transmitting an amount of 300 packets per test collection (i.e., 50 packets per SF per collection). The test series differed from each other for the heights of the transmitting antenna above sea level (see Section 5.2).

A circumstance that has to be verified is to check whether or not points A and B were in LoS. Hence, by resorting to Equation (6), by switching the transmitting antenna with the receiver one (see Figure 3) and by plugging into the Equation $h_{RX}$ in place of $h_{TX}$, it can be noticed that points A and B are always in LoS since the former is not behind the horizon because $D_{h}$ is bigger than *D*. Table 2 summarises what has just been described and it also contains the results computed by applying Equation (6).

At this stage, the concepts introduced in Section 4 can be applied and evaluated to the wireless link that has just been illustrated. First of all, the maximum radius of the first Fresnel zone $F_{1}$ can be calculated by applying Equation (5). Since a frequency diversity scheme was implemented by means of frequency hopping among the channels listed in Section 5.2, $F_{1}$ is evaluated by exploiting as carrier frequency the mean value of the ones previously listed (i.e., 867.8 MHz). The same convention holds for all the following Equations in which the frequency of the carrier is involved. Concerning the maximum height of the Earth bulge *H* amid the link endpoints, it can be derived by means of Equation (7). The theoretical free space loss $L_{FS}$ can be computed according to Equation (4), therefore an overestimation of the received signal power $P_{RX}$ can be obtained via Equation (3). Indeed, the latter is estimated by plugging into its Equation the values cited in Section 5.2 and Section 5.3 (i.e., $G_{TX}$, $G_{RX}$, $L_{TX}$ and $L_{RX}$), by exploiting the maximum power output for the transmitter $P_{TX}$ according to the regional regulations [33] (i.e., 14 dBm) and by considering a null value for miscellaneous losses $L_{M}$. Actually, there are several losses of that kind due to the fact that, as it will be seen in a while, the 60% of the first Fresnel zone is not free from obstacles. Unfortunately, though, the loss introduced by such a phenomenon is hardly numerically assessable. For the same reason, an overestimation of the link margin $L_{m}$ may be computed by exploiting Equation (2) bearing in mind that the Gateways shown in Section 5.3 have a sensitivity which varies along with the SF and the bandwidth. Indeed, according to the LoRaWAN concentrators datasheet [35] and considering that a bandwidth of 125 kHz was exploited throughout the tests, the Gateways sensitivity $S_{RX}$ spans from −137 dBm at SF=12 to −126 dBm at SF=7. Another consequence stemming from the presence of miscellaneous losses is that a lower value of $P_{RX}$, with respect to the one that would have been obtained from Equation (3), was truly experienced on average during the measurements thus entailing a narrower $L_{m}$. Notice that all of these quantities are independent of the set of tests since they only depend on the length of the link *D*. All the results stemmed out from the aforementioned Equations are reported in Table 3.

**Figure 8:**
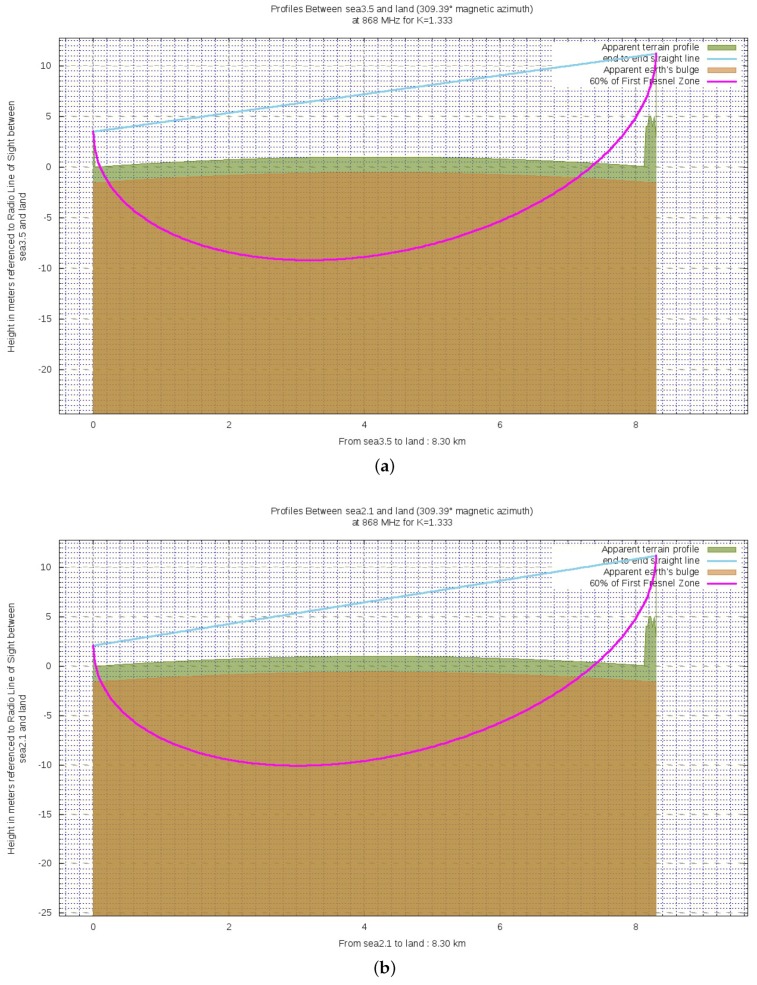
Ground profiles for either the exploited altitudes for the transmitting antenna: (**a**) 3.5 m; (**b**) 2.1 m.

The last condition to be assessed is the percentage of clearance the first Fresnel zone $F_{1_{C}}$. Indeed, despite the LoS status between the link endpoints, $F_{1}$ is partially occupied by the sea because of the Earth bulge. Figure 8 shows a qualitative report of the wireless link respectively when $h_{TX_{1}}$ is exploited (**a**) and $h_{TX_{2}}$ is used (**b**). Such images are obtained by making use of BotRf [36] which is a Telegram Bot that aids in the planning phase of wireless links. The cyan line represents the direct LoS path between the link endpoints while the magenta one represents the 60% of $F_{1}$. Finally, the brown area indicates the Earth curvature while the green one indicates the terrain profile. The reason for which the green area is for the most part flat is that it is actually occupied by the sea. Either the brown and green areas take into account the factor *k* (see Equation (Figure 4)) for the bending effect on radio waves due to the declining of atmospheric pressure. As it can be noticed, LoS is effectively achieved and the first Fresnel zone is densely occupied.

At this stage one can evaluate the percentage of clearance of the first Fresnel zone. Due to the fact that the transmitting and receiving antennas were installed at different heights, let us suppose that they shared the same altitude so as to ease the computation. In so doing, an overestimate of the clearance will be obtained. However, this is not an issue at all since such assessment has the mere scope of giving a flavour of the toughness of this specie of transmission over the sea. The maximum extent of the occupation of the first Fresnel zone by the sea due to the Earth bulge is experienced at the midpoint of the link since in the same location occur both of the maxima of $F_{1}$ radius as well as of the Earth lump. Such a percentage could be evaluated by firstly computing the distance between the sea level and the line connecting the link endpoints (i.e., *D* in Figure 2) at half of the link length *D* relying on the flat Earth model. This term can be computed as the sum of two factors: one is length of the minor cathetus belonging to the right triangle having half of the link length *D* as major cathetus and the transmitting antenna as the corner of its acute angle, and the other one is the height of the transmitting antenna $h_{TX}$. Secondly, the Earth curvature have to be taken into account by subtracting the maximum height *H* of the Earth bulge. Finally, this difference is the portion of $F_{1}$ free from obstacles. This procedure can be fulfilled via the Equation

(8) $$F_{1_{C}}=\frac{100\left[\left(\frac{h_{RX}-h_{TX}}{2}+h_{TX}\right)-H\right]}{F_{1}}.$$

The final step consists of plugging into Equation (8) the altitudes of the antennas for each trial group (see Table 2). The resulting values for $F_{1_{C}}$ are listed in Table 4.

It has to be underlined that the values within Table 4 are so scant owing to the fact that the radius of the first Fresnel zone $F_{1}$ is far longer than the antennas elevations. As a direct consequence, additional losses belonging to the miscellaneous class $L_{M}$ to be accounted for within the link budget (see Equation (3)) arise.

**Figure 9:**
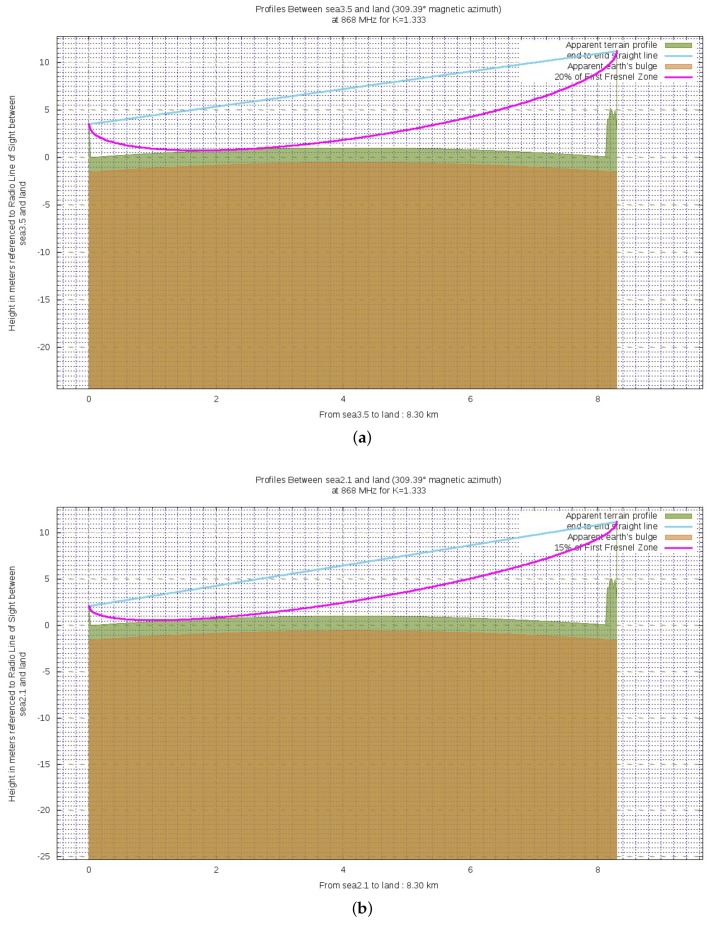
Finer estimates of the actual clearances for both the exploited altitudes for the transmitting antenna: (**a**) 20% for $h_{TX_{1}}=3.5$ m; (**b**) 15% for $h_{TX_{2}}=2.1$ m.

As it was previously said, figures within Table 4 are overestimates of the clearances of the first Fresnel zone. Indeed, by exploiting BotRf, it can be observed that finer estimates, though still rough, of the actual clearances are 20% for $h_{TX_{1}}$ and 15% for $h_{TX_{2}}$ (see Figure 9 bearing in mind that the same color convention of Figure 8 is observed).

Therefore, we deem we can label the whole tests setup as a worst case one (apart from the fact that a marine environment is itself a harsh environment) because of the following reasons:

- Antennas on both the sides were not installed by making use of overhead spots in order to limit the complexity related to their installation which was thought to be only a temporary one;
- As a direct consequence of the last point, only a limited clearance of the first Fresnel zone (far from being the 60%) was available;
- For the transmission the bigger CR was adopted which might had caused the loss of some packets due to the inability of the Gateways to restore corrupted data.

## 6. Results and Discussion

At first, each of the test groups will be in detail dissected. Then, a general analysis on the whole measurements campaign will be provided. A commonly examined outcome is the percentage of received packets and a solution to augment such a rate is to decrease the CR because some of the missing packets could be so due to the inability to restore corrupted data at the receiver side. However, the maximum CR was selected for the measurements campaign so as to implement a worst case scenario.

### 6.1. Group Specific Discussion

Hereinafter, the RSSIs and the SNRs will be analyzed, for each SF and for each test trial, by modeling them as discrete random variables. Indeed, the Gateways sample those quantities in a discrete fashion. In particular, the RSSIs take values in the set

R={−137,−136,⋯,−1,0}

where the elements of R are expressed in dBm, while the SNRs take values in the set

S={−20.0,−19.9,⋯,19.9,20.0}

where the elements of S are expressed in dB. Since the measured RSSIs at the Gateways side can be considered as the power of the received signals, a comparison between the values of RSSIs that were recorded during the tests and the value of $P_{RX}$ reported in Table 3 can be accomplished. The former ones were lower than the latter due to either the limited clearance of the first Fresnel zone (see Table 4) and to the fact that the sea behaves as a huge ground plane because of the salinity of the water. Such phenomena cause a difference between the theoretical values and the measured ones of slightly more than 20 dBm. However, this did not notably affect the performance of the wireless communication link. Along with those variables, also the percentage of the received packets for each SF for each test group will be evaluated.

#### 6.1.1. Group #1

**Figure 10:**
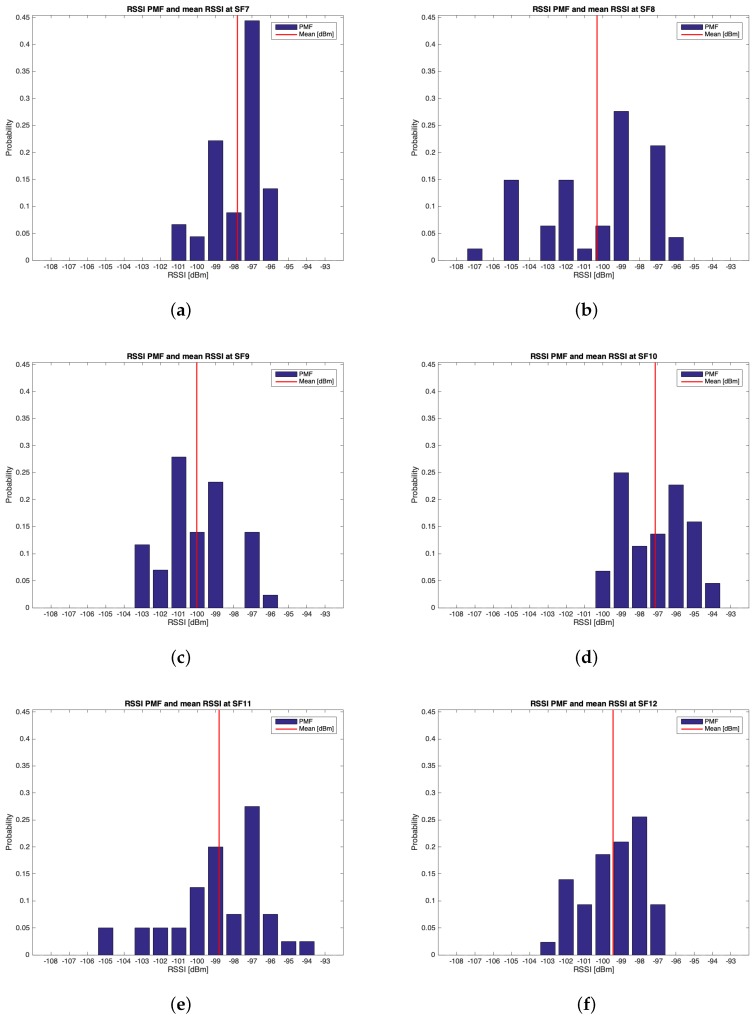
Group #1 RSSIs PMFs: (**a**) SF=7; (**b**) SF=8; (**c**) SF=9; (**d**) SF=10; (**e**) SF=11; (**f**) SF=12.

**Figure 11:**
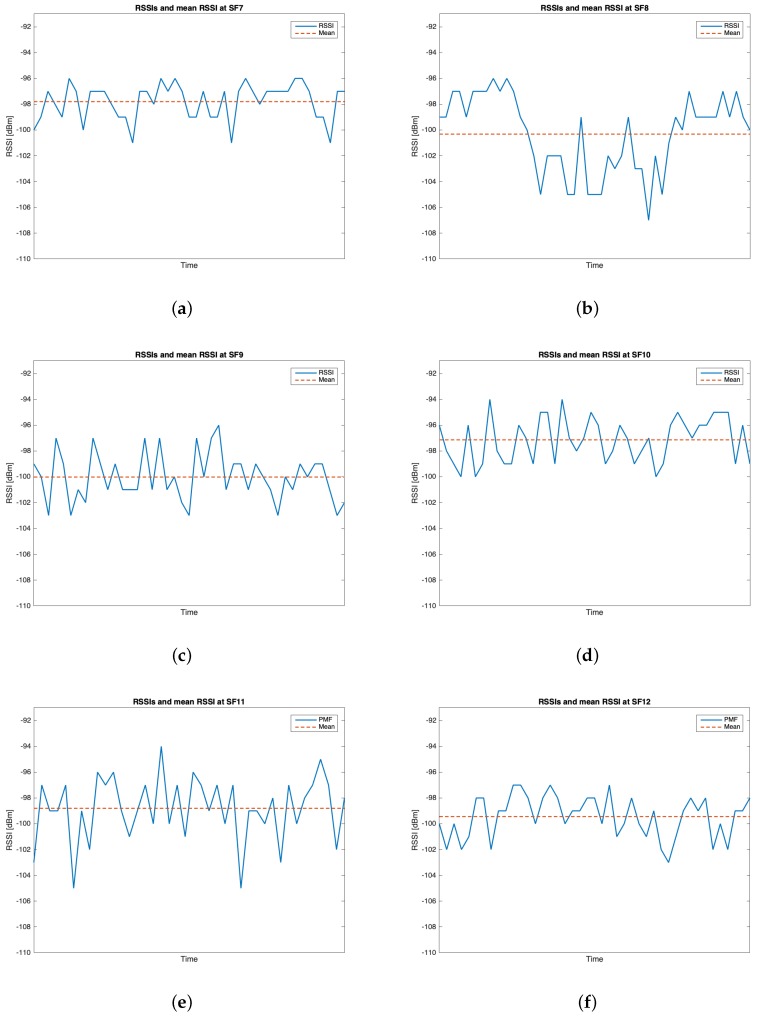
Group #1 RSSIs temporal trend: (**a**) SF=7; (**b**) SF=8; (**c**) SF=9; (**d**) SF=10; (**e**) SF=11; (**f**) SF=12.

**Figure 12:**
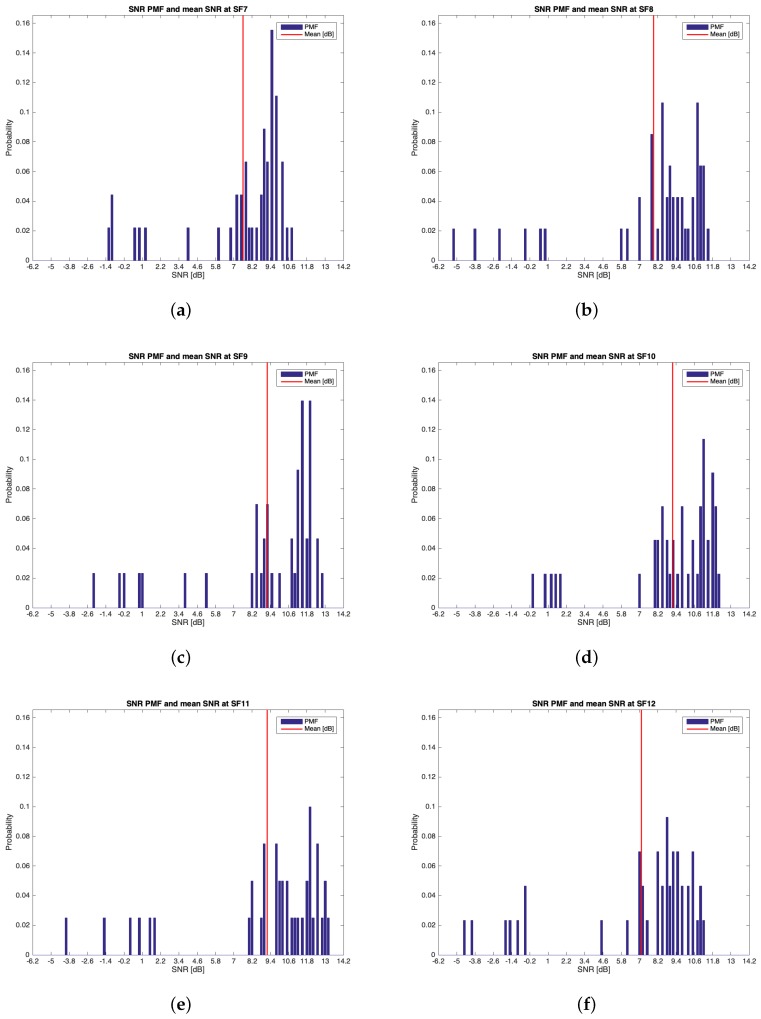
Group #1 SNRs PMFs: (**a**) SF=7; (**b**) SF=8; (**c**) SF=9; (**d**) SF=10; (**e**) SF=11; (**f**) SF=12.

**Figure 13:**
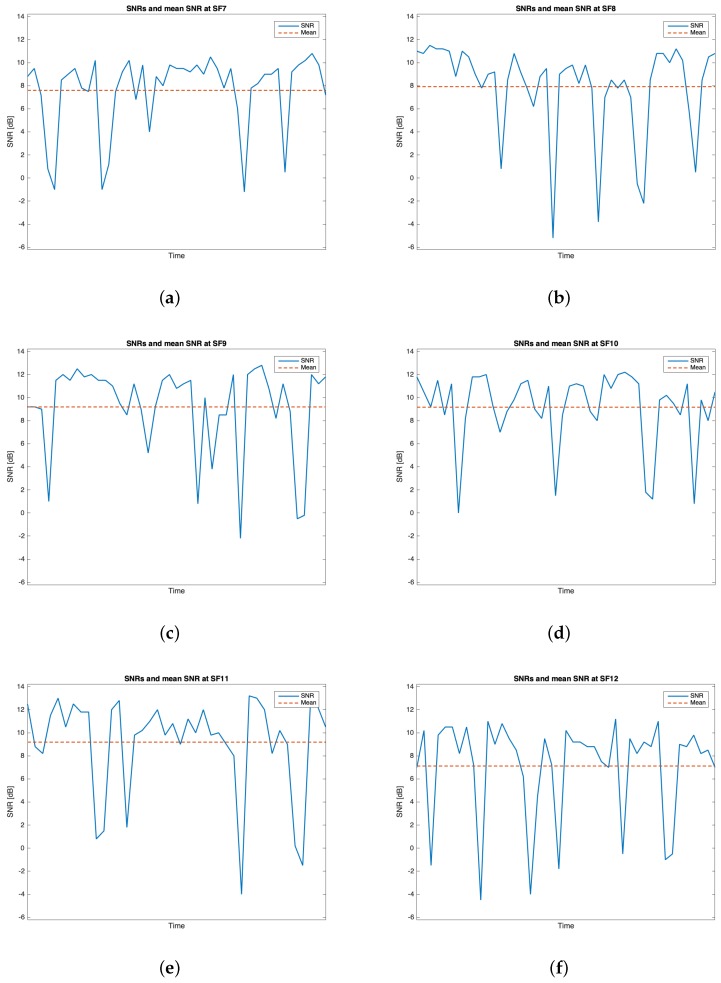
Group #1 SNRs temporal trend: (**a**) SF=7; (**b**) SF=8; (**c**) SF=9; (**d**) SF=10; (**e**) SF=11; (**f**) SF=12.

The characteristics of this measurements group are reported in Table 2. During this setup, 262 packets out of the 300 transmitted were received (i.e., the 87.33%). Table 5 reports the mean values, the standard deviations of the RSSIs and SNRs and the number of received packets of out 50 for each SF. It can be noticed that the maximum mean values for RSSIs and for SNRs were respectively observed for SF=10 (i.e., $\mu_{RSSI}=-97.136$ dBm) and for SF=9 (i.e., $\mu_{SNR}=9.193$ dB). However, due to the minimum difference between the mean RSSIs of SF=7 and SF=10 and since the standard deviation associated to the former is less than the one of the latter, it is reasonable to claim that SF=7 was the most suitable SF during this measurements setup. Figure 10, Figure 11, Figure 12 and Figure 13 in turn show the RSSI Probability Mass Function (PMF), the RSSI temporal trend, the SNR PMF and the SNR temporal trend dividing them by SF. Regarding the latter two, some negative SNRs can be spotted underlining the capability of LoRa to demodulate heavy noisy signals. Focusing on Figure 11 and Figure 13 a lack of values in the horizontal axis can be noticed. This is due to the fact that their purpose is just to display how the RSSIs and the SNRs varied during the tests. Since this test group turned out to be successful, it was chosen to worsen the link by lowering down the transmitting antenna to $h_{TX_{2}}$ (see Table 2) for the next sets of trials.

#### 6.1.2. Group #2

**Figure 14:**
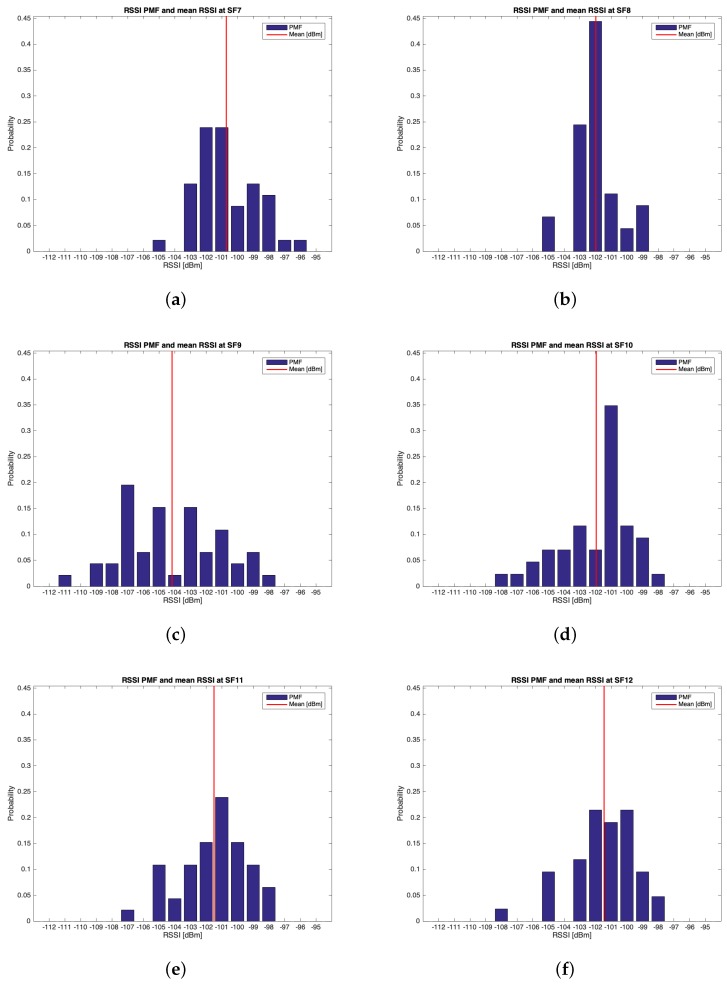
Group #2 RSSIs PMFs: (**a**) SF=7; (**b**) SF=8; (**c**) SF=9; (**d**) SF=10; (**e**) SF=11; (**f**) SF=12.

**Figure 15:**
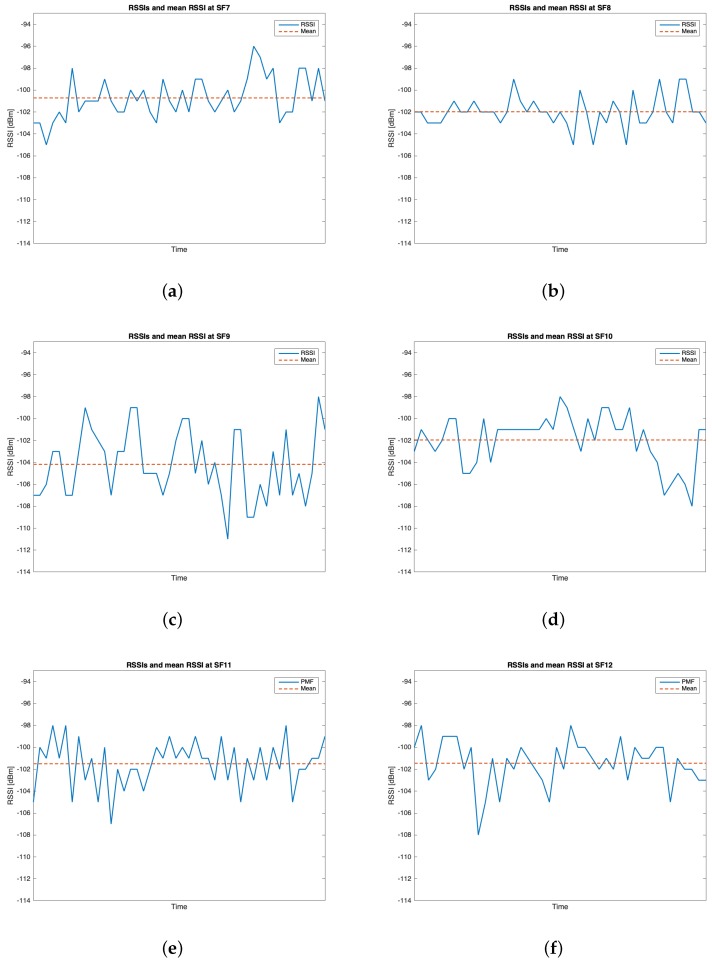
Group #2 RSSIs temporal trend: (**a**) SF=7; (**b**) SF=8; (**c**) SF=9; (**d**) SF=10; (**e**) SF=11; (**f**) SF=12.

**Figure 16:**
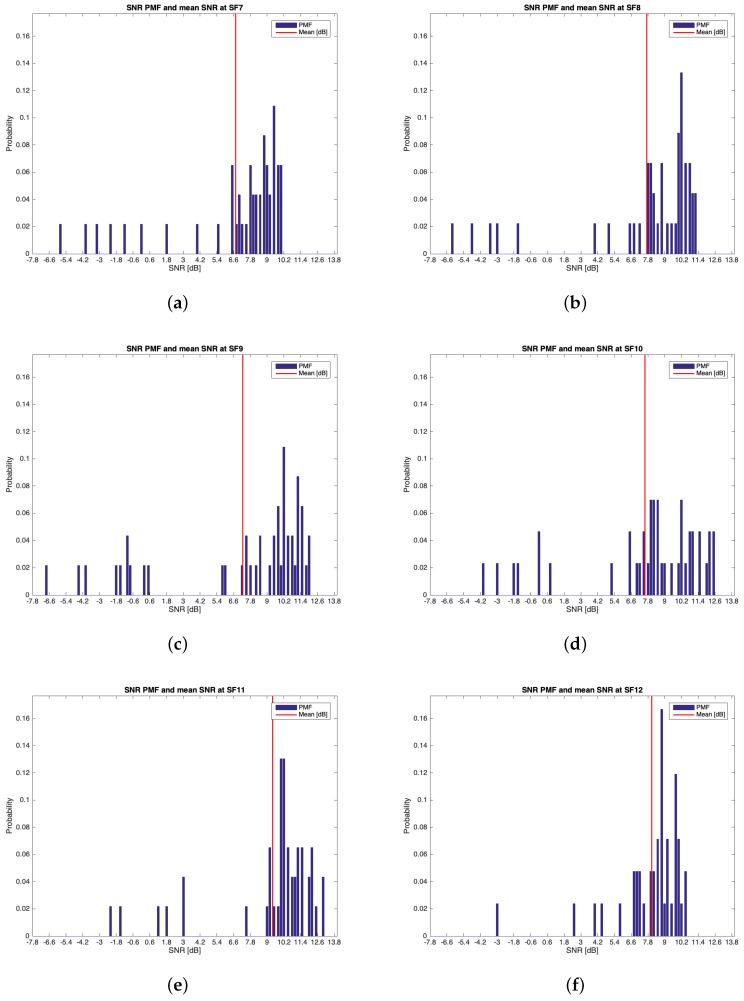
Group #2 SNRs PMFs: (**a**) SF=7; (**b**) SF=8; (**c**) SF=9; (**d**) SF=10; (**e**) SF=11; (**f**) SF=12.

**Figure 17:**
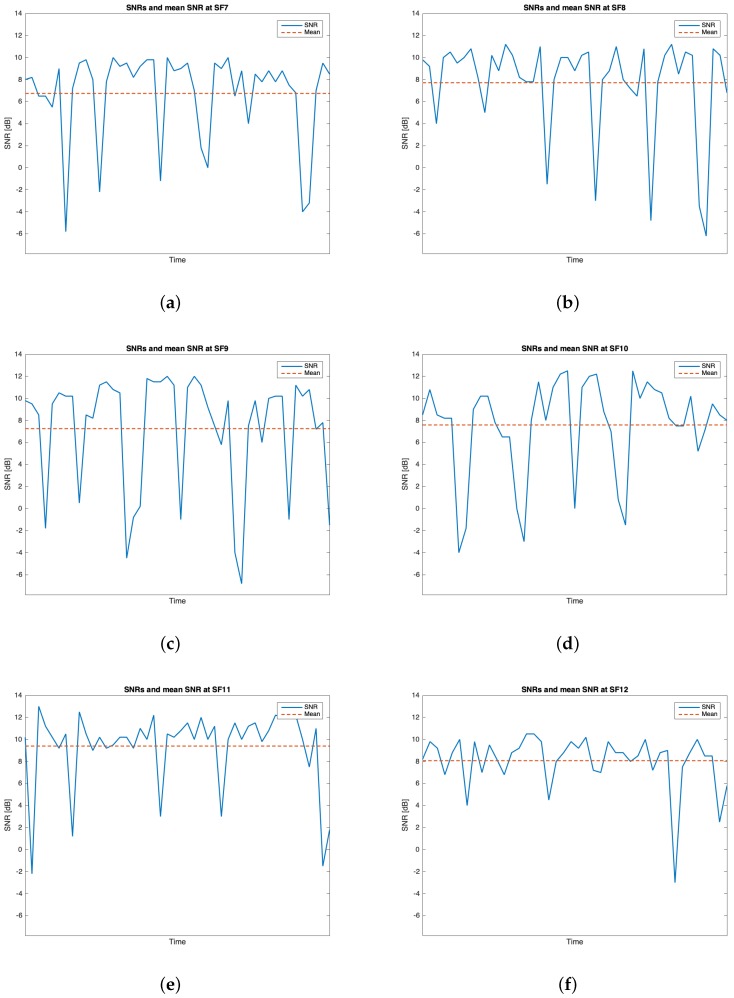
Group #2 SNRs temporal trend: (**a**) SF=7; (**b**) SF=8; (**c**) SF=9; (**d**) SF=10; (**e**) SF=11; (**f**) SF=12.

This trial set was characterised by the data listed in Table 2. Throughout this group of experiments, 268 packets out of the 300 transmitted were received (i.e., the 89.33%). Table 6 outlines the mean values, the standard deviations of the RSSIs and SNRs and the number of received packets out of 50 for each SF. The best RSSI mean value was experienced for SF=7 (i.e., $\mu_{RSSI}=-100.717$ dBm) while the best SNR mean value was observed for SF=11 (i.e., $\mu_{SNR}=9.396$ dB). However, because of the limited difference amid the mean RSSIs of SF=7 and SF=8 and due to the fact that the latter has the smallest standard deviation, it is plausible to assert that SF=8 is the one having the best performances throughout this trial setup. Figure 14, Figure 15, Figure 16 and Figure 17 respectively show the RSSI PMF, the RSSI temporal trend, the SNR PMF and the SNR temporal trend dividing them by SF. As it was averred for the analysis of group #1, Figure 15 and Figure 17 do not present any value in the horizontal axis since their purpose is just to visualize how the RSSIs and the SNRs varied during the tests. It can be seen that the number of the negative values for SNR was increased with respect to the ones belonging to group #1.

### 6.2. Overall Discussion

With the aim of accomplishing a finer statistical analysis, it would have been necessary to collect a greater number of packets. Sadly, though, the low data-rate and the limitations on the duty cycle due to the spectrum usage regulations hindered the measurements. Despite it, we deem that the presented method and results could be valuable to render insights into the potentialities of a LoRaWAN network to be employed in a marine environment. Indeed, for the purposes of the application scenario, the feasibility of the LoRaWAN communication ashore was successfully proven because of the total amount of 600 packets that were transmitted throughout the trial groups, 530 were correctly received and demodulated (i.e., roughly the 88.3%). Moreover, considering the distribution of the RSSIs portrayed in the relative Figures in Section 6.1, there was enough link margin (i.e., from 25.55 dBm at SF=7 to 36.55 dBm at SF=12 on average) to exploit worse wireless link setups. Some instances are further lowering down the antennas elevations thus reducing their installation complexity, diminishing the transmitter power output thus augmenting the end node battery lifetime or, even though it is not required by the system requirements, covering longer distances.

Due to the nature of the application scenario, a more complete analysis on the performance of the communication ashore would have been carried out by considering the behavior of the system whenever different weather conditions, with respect to the one experienced during the measurement campaign, will have to be faced. As it was claimed earlier on, such trials were impossible to be actually analysed due to a logistical constraint associated with the boat we employed to reach the breeding cages. Nevertheless, we do not expect significant path losses caused by meteorological events by virtue of what is reported in the sixth chapter of Seybold book [34]. It presents several losses induced by atmospheric effects by showing their models and graphical representations to be taken into account during the design of a wireless link. Moreover, it also points out remedies.

Coastal and marine environments are particularly susceptible to fades due to atmospheric multipath and the preferred solution to mitigate such a shortcoming for fixed-length links is to establish an antenna diversity scheme. Hence, this strategy was adopted by employing two antennas at the receiver side.

Atmospheric attenuation due to gaseous absorption (e.g., oxygen and water vapor) is not significantly detrimental for sub-GHz wireless links. Indeed, Seybold [34] reports plots showing the specific atmospheric attenuation of standard atmosphere and of dry air in function of the frequency for a horizontal path (which can be assumed as the one we presented beforehand). In both the cases, the specific attenuation is less than 0.01 dB/km thus making it negligible for our application.

Apart from the gaseous one which has just been treated, there exist two forms of water that affect wireless propagation: precipitation (e.g, rain and snow), and suspended water droplets forming clouds and fog. Snow is directly related to the moisture content of the particles within the air but the effect of its attenuation is relatively smaller with respect to the one due to rain. The latter attenuation is deeply discussed in the tenth chapter of Seybold book [34] which concludes that the extent of this kind of loss is less than 0.01 dB/km for sub-GHz applications. On the other hand, clouds and fog rarely contribute to the detriment of a wireless link.

Similar considerations may be drawn for what concerns possible antennas orientation mismatches due to the fact that the transmitting one is expected to be mounted on top of a seamark buoy. Since the tests were performed using a small dimensions boat with not perfect wave conditions, as stated in Section 5.4, a certain degree of motion of the vessel was present: nevertheless this did not significantly affect the performances of the link. While no analytical study was carried out due to the random nature of the movement of the boat, a set of tests with different angles of orientation may be performed in future works.

#### Groups Comparison

**Figure 18:**
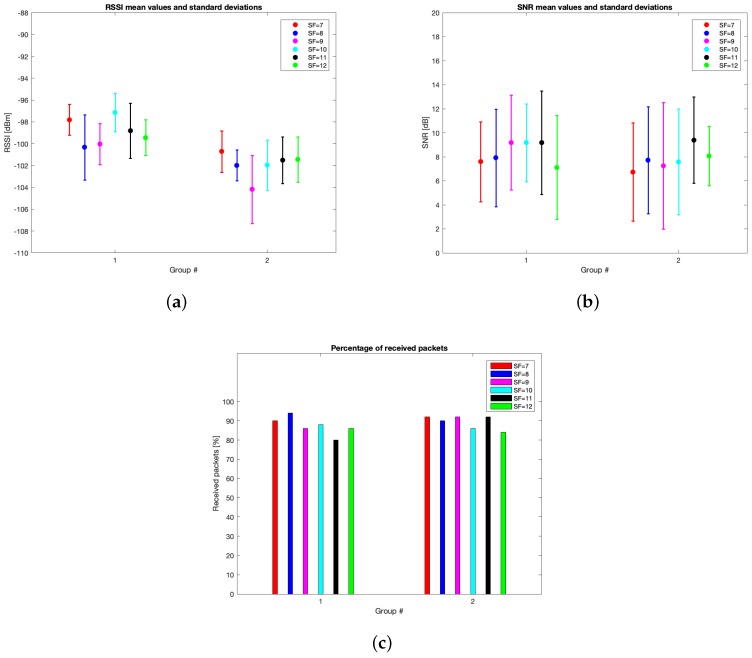
Group comparison graphical analysis divided by SFs: (**a**) RSSIs mean values and standard deviations; (**b**) SNRs mean values and standard deviations; (**c**) percentage of received packets.

Figure 18 shows the comparison between the mean values and the associated standard deviations for the RSSIs and SNRs divided by SF and test groups. Furthermore, the same Figure displays the percentage of received packets split by SF and test trials.

After the transmission tests that have just been illustrated, the setup for the communication ashore of the needed information has to be identified. First of all, an optimal strategy for the receiving antennas installation is their placement as high as possible since the transmitting one cannot be mounted any higher than the upper extremity of the seamark buoy. Indeed, in spite of the fact that the limited employed elevations for the Gateways antennas throughout these transmission tests ensured good performances, it is advisable to install higher those devices so as to better exploit the first Fresnel zone thus increasing the rate of correctly received uplinks from end nodes. However, a trade off between the installation costs, its complexity and the altitude at which the Gateways antennas are placed has to be reached. As it was mentioned earlier on, another approach to augment the percentage of received packets is to make use of smaller CRs thus reducing the probability that the Gateways cannot demodulate signals due to corrupted data. But, as a drawback though, longer packets would be broadcast hence increasing the relative Time on Air (ToA) and the end node power consumption as well. Finally, despite it is not theoretically suitable for long distance links, according to the aforementioned trial groups SF=7 is the most favourable SF to convey the sampled data from offshore towards ashore due to several reasons:

- Such a SF provided acceptable rates of received packets even though it was not the best one;
- In both groups #1 and #2 it turned out in having some of the best RSSI mean values and standard deviations, as well as satisfactory SNR statistics. While the differences among the RSSI values achieved using the different SFs can be attributed more to statistic fluctuations rather than to actual better performances, mostly due to the small number of transmitted packets, it is however possible to state that in the proposed conditions the performances of the six SFs are almost comparable, and do not suggest any particular choice in this sense;
- Its most important feature for the application scenario is that it is the SF ensuring the shortest ToA for the same packet length thus reducing power requirements.

## 7. Conclusions

The aim of this paper was to demonstrate the feasibility of a LoRaWAN network to be used for data collection in marine environments, with the transmitting device placed in the middle of the sea and the Gateway placed ashore. The proposed network architecture was expected to be used for the monitoring of offshore sea farming plants, within the framework of the *SeaFactory* project, a research and development project financed by Tuscany Region, Italy.

A fully operating network prototype was set up and then validated by sorting out a measurements campaign, that was held in the production area of an aquaculture partner company, from offshore towards land. The distance between the offshore breeding cages and the plant belonging to the firm (i.e., 8.33 km) was successfully covered thus proving the feasibility of the link. In addition, the tests made evident that the best SF to be exploited during the future installation of the whole system is SF=7 since it ensured limited packets losses, satisfactory mean values and standard deviations either for RSSIs and SNRs and the minimum power consumption compared with higher SFs for the same packet length. Antenna elevation did not significantly diminish the received packets rate. However, with the aim of increasing such a proportion the antennas should be placed at higher elevations along with the adoption of smaller CRs so as to reduce the probability that Gateways cannot demodulate signals due to corrupted data.

## Figures and Tables

**Table 1:** Coordinates of points A and B of Figure 7.

Point	Latitude [°]	Longitude [°]
A	42.895494	10.629385
B	42.942858	10.550552

**Table 2:** Antennas heights, exploited SFs, number of transmitted packets and distance of the horizon from the receiver antennas for each trial group.

Group	$h_{\mathrm{TX}}$ [m]	$h_{\mathrm{RX}}$ [m]	SFs	Transmitted Packets	$D_{h}$ [km]
#1	3.5	13.2	7÷12	300	12.969
#2	2.1	13.2	7÷12	300	12.969

**Table 3:** Values of the quantities introduced in Section 4.

Quantity	Value
$F_{1}$	26.818 m
*H*	1.021 m
$L_{FS}$	109.631 dB
$P_{RX}$	−78.791 dBm
$L_{m}$	58.209÷47.209 dBm
*D*	8.33 km

**Table 4:** Percentages of clearance of the first Fresnel zone for each test group.

Group	$F_{1_{C}}$ [%]
#1	27.33
#2	24.72

**Table 5:** Experimental results from group #1.

SF	$\mu_{\mathrm{RSSI}}$ [dBm]	$\sigma_{\mathrm{RSSI}}$ [dBm]	$\mu_{\mathrm{SNR}}$ [dB]	$\sigma_{\mathrm{SNR}}$ [dB]	Received Packets
7	−97.800	1.408	7.604	3.329	45
8	−100.319	2.986	7.923	4.059	47
9	−100.023	1.883	9.193	3.935	43
10	−97.136	1.747	9.171	3.245	44
11	−98.800	2.524	9.191	4.312	40
12	−99.442	1.637	7.121	4.326	43

**Table 6:** Experimental results from group #2.

SF	$\mu_{\mathrm{RSSI}}$ [dBm]	$\sigma_{\mathrm{RSSI}}$ [dBm]	$\mu_{\mathrm{SNR}}$ [dB]	$\sigma_{\mathrm{SNR}}$ [dB]	Received Packets
7	−100.717	1.893	6.744	4.093	46
8	−101.978	1.406	7.716	4.446	45
9	−104.174	3.115	7.252	5.275	46
10	−101.954	2.309	7.586	4.413	43
11	−101.500	2.127	9.396	3.586	46
12	−101.452	2.074	8.074	2.464	42
